# A Rare Case of Multiorgan Calciphylaxis in a Patient with Stage 5 Chronic Kidney Disease

**DOI:** 10.1155/2018/9603680

**Published:** 2018-10-18

**Authors:** Abdulrahman Ahmad, Ali Albaghli, Adel Michael, Khaled Refaat, Mohammad Omar, Ahmad Ibrahim, Bahaa Elmenshawy, Ashraf Maher, Rasha Alramah, Sami Mikhail, Mustafa Almahmid, Husain Alenezi, Yahya Elshebiney

**Affiliations:** Department of Urology, Aladan Hospital, Kuwait

## Abstract

Calciphylaxis or calcific uremic arteriolopathy (CUA) is a potentially life-threatening vasculopathy involving the skin and subcutaneous tissues. It is usually associated with chronic kidney disease (CKD) and rarely with acute renal failure or predialysis patients. The clinical diagnosis of calcific uremic arteriolopathy relies on high index of suspicion. CUA is commonly associated with secondary hyperparathyroidism and high serum calcium and phosphate products. Moreover, using biopsy as a diagnostic tool is controversial, due to the high risk of poor wound healing and sepsis. Radiological studies usually reveal extensive calcification of branching vessels such as penile arteries, eventually leading to gangrene formation in extremities and penis. Histopathological analysis confirms the diagnosis of calcific uremic arteriolopathy and rules out the presence of malignancy. CUA is a systematic disease that involves multiple organs, and to the best of our knowledge this is the first reported case involving the penis, bladder, and eyes.

## 1. Introduction

Calcific uremic arteriolopathy can be characterized by obstructive vasculopathy, with calcification of small arteries and arterioles resulting in luminal occlusion and subsequently cutaneous necrosis [1]. The incidence of CUA is estimated to be approximately 1% in patients with CKD and 4% in patients on dialysis [2]. The diseases carry a bad prognosis with a mean time to death of 2.5 months [3]. CUA usually involves area in the thighs and buttocks and also affects distal phalanges of the hands and feet. Rare systemic manifestation includes ischemia and infarction of the bowel, myocardium, brain, optic nerve, and muscles [4]. Diagnosis is based on patient presentation, clinical signs, and blood investigations.

In addition, tissue biopsy can be used to differentiate CUA from similar conditions such as diabetic vascular disease, purpura fulminans, atheroembolic disease, antiphospholipid antibody syndrome, peripheral artery disease, vasculitis, and necrotizing infections [5]. Due to patients poor wound healing and potential risk of developing sepsis, biopsy is not considered essential for diagnosis [6]. Histopathological findings are calcification in the vascular media, intimal inflammation and hyperplasia, obliterative endovascular fibrosis and microthrombi in small and medium-sized vessels of the skin, and subcutaneous tissue leading to necrosis of dermal, subdermal, and adipose tissues [4]. Treatment of CUA includes conservative management and surgical debridement. In our case, we started with conservative therapy, but due to intolerable pain, we proceeded with partial penectomy.

## 2. Case Presentation

**Figure 1:**
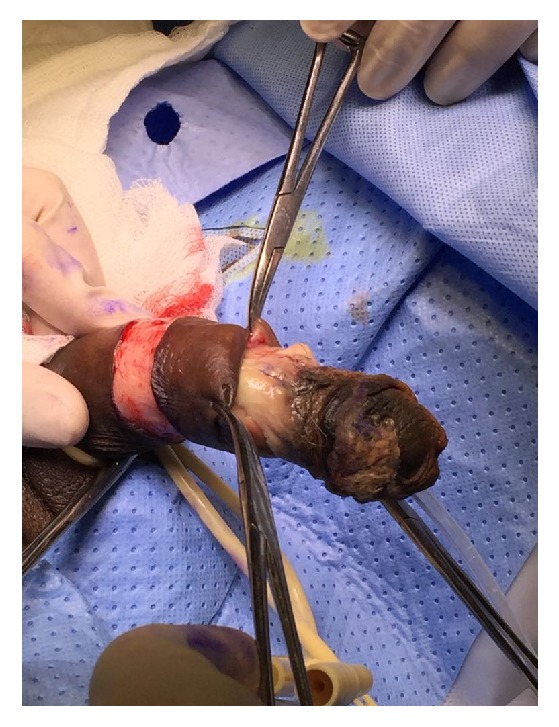
Preoperative appearance of the penis.

A 60-year-old gentleman presented in clinic complaining of dysuria and intermittent painless hematuria and severe penile pain. His comorbidities include stage 5 chronic kidney disease, peripheral vascular disease, and insulin dependent diabetes mellitus. The patient denies history of trauma, and there was no evidence of vitamin D deficiency or thrombophilia. On examination, he had a tight meatus, blackish discoloration of the tip of the glans, and tender hard gangrenous mass of the glans (Figure 1), which was proven to be a calciphylaxis gangrene by histopathological assessment.

**Figure 2:**
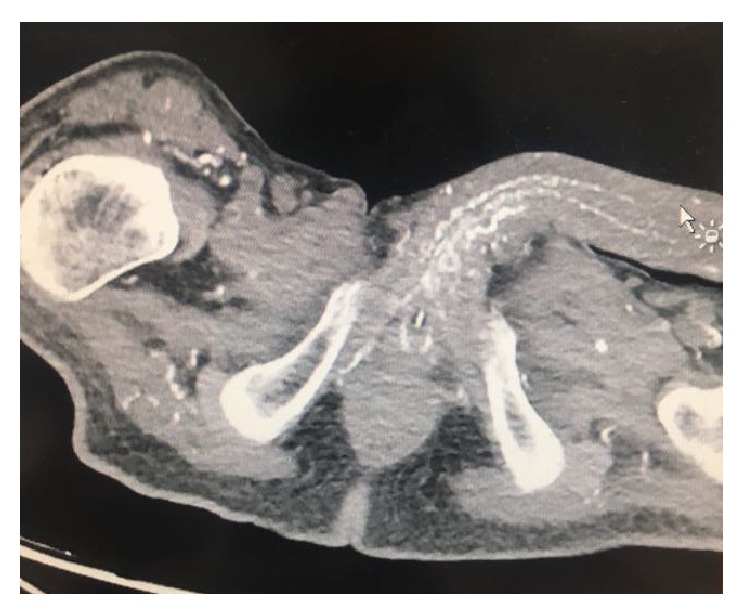
Calcification of the penile vessels and tissues.

**Figure 3:**
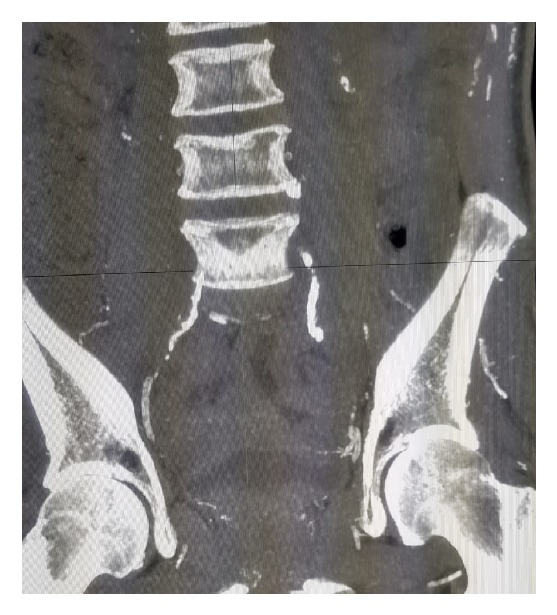
Bilateral obstructive calcification of internal illiac vessels.

Laboratory results revealed mildly elevated inflammatory markers including ESR and PCT. Fasting blood sugar was 12.8 mmol/L on admission and then was controlled and reached 5.5 mmol/L. Serum calcium was normal 2.53 mmol/L, and serum phosphate was also normal 1.4 mmol/L, giving a high calcium phosphate product of 75.9 mg/dL (normal range: 20.6–52.5 mg/dL). In addition, parathyroid hormone level was persistently elevated 70 pg/mL (N-terminal: 8 to 24 pg/mL). Albumin was 40 g/L. Due to the history of hematuria, CT urography was done and it showed extensive calcification of the corpus cavernosa, penile vessels, and soft tissues (Figure 2), obstructive calcified of bilateral internal iliac vessels both anterior and posterior branches (Figure 3).

**Figure 4:**
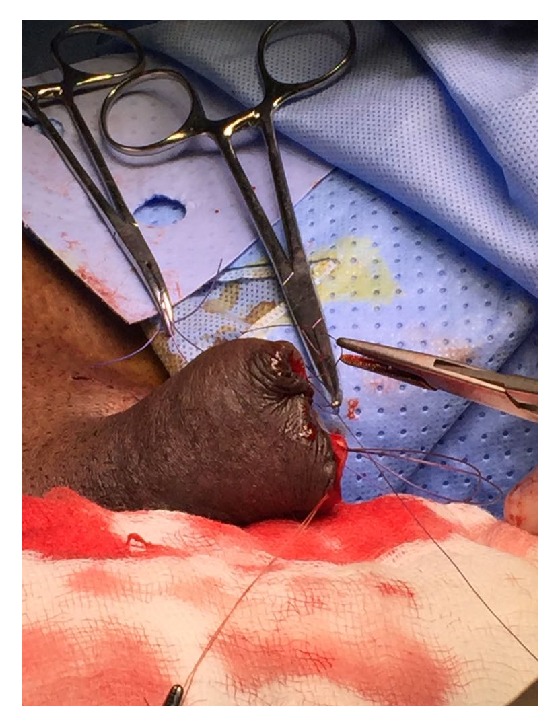
Partial penectomy.

**Figure 5:**
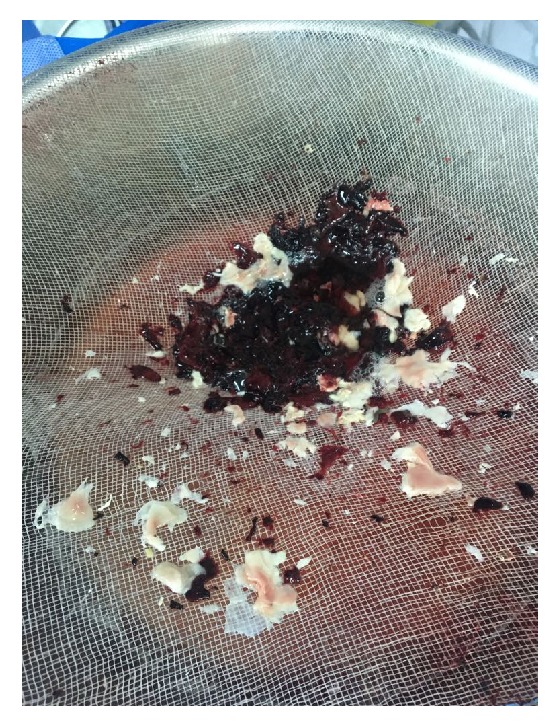
Clots and sloughed necrotic tissues evacuated from the bladder.

Conservative therapy was initiated in form of wound debridement, systemic antibiotics and sodium thiosulfate, and tight blood sugar control, but due to severe penile pain we proceeded with partial penectomy (Figure 4). Additionally, a cystoscopy was done and showed sloughed necrotic bladder wall and diffuse hematuria uncontrolled by fulguration (Figure 5). Postoperatively, he developed sepsis with persistent hematuria and was shifted to intensive care unit (ICU) for resuscitation. Sepsis parameters improved in the ICU. Trail of ALUM and dicynone instillation were unsuccessful in controlling the hematuria, so the decision for redo cystoscopy was made, and we found a diffuse uncontrollable bladder wall bleeding; therefore bilateral internal iliac angioembolization was done and it was successful in controlling the hematuria, leading finally to Hemodynamic stability of the patient. Histopathology confirmed the diagnosis of calcific uremic arteriolopathy of the penis, and bladder biopsy showed diffuse blood vessels with no evidence of malignancy.

After being discharge he presented to the clinic with sudden onset of left eye blindness. Magnetic resonance angiography (MRA) of the brain demonstrated the presence of multiple lacunar infarcts and inflammatory changes in the left optic nerve, consistent with optic nerve ischemia or inflammation. The MRA also showed multiple areas of bilateral narrowing of ACA and MCA arteries and none of the ophthalmic arteries were visualized.

## 3. Discussion

Calcific uremic arteriolopathy is not well understood, with multifactorial aetiologies. Uremia creates an inflammatory reaction that further suppresses calcification inhibitors. In previously reported cases, 76% of patients with penile necrosis secondary to calcific uremic arteriolopathy have concurrent diabetes mellitus, compared to 39% of ESRD patients, which could suggest that diabetes is a predisposing factor [7]. Two-thirds of patients with penile calcific uremic arteriolopathy have extragenital gangrenous lesions [7], as demonstrated in our patient who had involvement of the eyes, bladder, and upper and lower limbs. Other risk factors that were linked with calcific uremic arteriolopathy include female gender, mineral and bone disorders, obesity, warfarin anticoagulation, and Caucasian ethnicity [8].

Diagnosis is usually made from clinical presentation, metabolic parameters, and imaging. A potential diagnostic indicator is a calcium phosphate product over 70 mg/dL, as it has been shown that those with penile calcific uremic arteriolopathy had a significantly higher calcium phosphate product than a control group of patients with ESRD (*p* < 0.05) [7]. Radiological studies used in diagnosis include penile Doppler ultrasound, CT scan, and MRI. CT scan is the most sensitive modality to assess the extent of vascular and soft tissue calcification, necrosis, and infection with detecting the presence of air in the affected tissues. Biopsy has been used in the past for diagnosis; however recently it has been discouraged because it carries a high risk of poor wound healing, sepsis, and necrotic spread. In our case we were not able to obtain a histopathological diagnosis of calciphylaxis in the eye and bladder; instead we depended on the clinical picture, metabolic workup and radiological findings. First-line therapy is conservative management and it includes aggressive wound care and systemic antibiotics, and the most promising of them all is the use of sodium thiosulfate. Sodium thiosulfate has been shown to successfully treat calcific uremic arteriolopathy with clinical improvement within 2 weeks and complete resolution of pain after 8 months of treatment [9]. Second-line therapy includes surgical management with total or partial penectomy.

## 4. Conclusion

Calciphylaxis is a rare systemic disorder that may involve multiple organs and mostly relies on a high index of clinical suspicion for diagnosis. The aim of reporting this case was to raise awareness of the condition among urologists and to broad their differential diagnosis when reviewing a penile lesion in a patient with positive risk factors. Early detection of the disease increases the chance of clinical improvement and disease resolution and improves quality of life.
